# Salinity–Sediment Interactions Governing CO_2_ Hydrate Formation, Kinetics and Stability in Marine Environments

**DOI:** 10.1021/acsomega.6c02301

**Published:** 2026-05-01

**Authors:** Beatrice Castellani

**Affiliations:** Department of Engineering, 9309 University of Perugia, via G. Duranti 25, Perugia 06125, Italy

## Abstract

The storage of carbon dioxide in the form of gas hydrates represents a promising strategy for long-term CO_2_ sequestration in marine environments. This work investigates the combined effects of salinity and natural sand on the formation, dissociation and stability of CO_2_ hydrates under conditions representative of marine sediments. A systematic experimental comparison was carried out between binary gas–water systems and three-phase gas–water–sand systems, with and without salinity (3 wt % NaCl), at two initial pressures (25 and 35 bar) using a 1 L high-pressure reactor. The natural sand was sampled from the Adriatic Sea. It is formed mainly by quartz and is primarily mesoporous with limited microporosity. The particle size is mainly included in the range of 100–200 μm. The introduction of natural sand significantly mitigates salinity-induced inhibition, particularly at higher pressure, increasing hydrate density from 100.7 to 166.6 g_CO2_/m^3^ and water utilization from 24.7% to 40.9% in saline systems. Kinetic analyses demonstrate that pressure controls the overall formation rate. At low pressure, sand acts mainly as an enabling factor in saline systems, while at high pressure, it induces a transition from continuous bulk growth to pore-controlled formation. Dissociation kinetics reflect the formation history and hydrate morphology. Salinity primarily controls the dissociation rates, while sand redistributes hydrate within the pore space, modifying the temporal pattern of CO_2_ release. Equilibrium measurements indicate that natural sand shifts hydrate stability toward less restrictive conditions and partially compensates for salinity inhibition for the investigated grain-size range.

## Graphical abstract

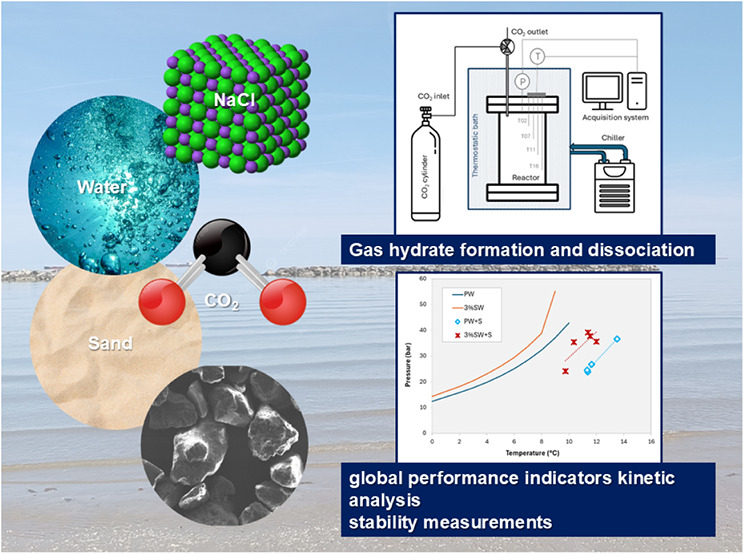

## 1 Introduction

Carbon capture and storage (CCS) is considered a crucial strategy for global climate mitigation. The Intergovernmental Panel on Climate Change (IPCC) emphasizes that nearly all pathways compatible with limiting warming to 1.5 °C require large-scale deployment of CCS.^1^ Similarly, the International Energy Agency (IEA) identifies CCS as a critical pillar of its Net Zero by 2050 scenario, calling for the capture of gigatonnes of CO_2_ annually to decarbonize existing energy and industrial systems and to support low-carbon hydrogen production.^2^

Despite there being more than 700 CCS projects under development, implementation remains far below the required levels. Current global announcements cover around 60% of the required storage capacity for 2030.^3^

Thus, the transition to a net-zero scenario fundamentally depends on the rapid development of CO_2_ storage systems capable of ensuring permanent sequestration over geological time scales. Geological storage of CO_2_ typically relies on saline aquifers (deep brine formations), depleted oil and gas reservoirs, unmineable coal seams, and rocks for mineralization.^4,5^ Among the viable geological media, saline aquifers have the greatest theoretical storage capacity (10000 Gt CO_2_), while depleted oil and gas reservoirs contribute more modest volumes (10–100 Gt CO_2_).^6,7^ Unmineable coal seams play a limited role in geological CO_2_ storage, while basaltic formations provide an alternative pathway based on in situ mineral carbonation.^8^

They are all constrained in practice by structural and geochemical limits. In fact, recent assessments indicate that the world’s geologically “safe” CO_2_ storage potential likely lies near 1500 Gt CO_2_, underscoring the vast but not unlimited capacity of subsurface systems.^9^

Recently, it has been proposed to store CO_2_ in marine environments through the formation of CO_2_ hydrates, either within subseafloor sediments or directly at the seabed.^10^ Gas hydrates are crystalline compounds composed of water molecules that form cages around gas molecules, in this case, CO_2_.^11^ They are thermodynamically stable under high pressure (tens of bars) and low temperature (some degrees above 0 °C). Under these conditions, the hydrate phase is favored over free gas or liquid water. One cubic meter of CO_2_ hydrate is capable of storing about 164 m^3^ of CO_2_ at standard temperature and pressure.^11^

The concept of storing CO_2_ directly in marine environments as gas hydrates originates from natural processes. In fact, gas hydrates have existed in nature for millennia as natural gas hydrates (NGH), which represent an important unconventional gas resource, exhibiting long-term stability within marine sediments.^12^ A previous line of research focused on CO_2_ storage through replacement in NGH; however, limitations in process efficiency and the dependence of the replacement on the complex interplay between water, sediment, and the involved gas species have motivated the investigation of direct CO_2_ hydrate storage, also because CO_2_ hydrates form under even more favorable thermodynamic conditions.^13,14^

The formation of CO_2_ hydrates within sediments can immobilize the injected CO_2_ by converting it into a solid phase, reducing its mobility.^15–17^ In hydrate-free sediments, the formation of CO_2_ hydrates can also enhance structural integrity by acting as a bonding agent between grains.^18^ Experimental studies show that the presence of CO_2_ hydrates increases the dynamic Young’s modulus by up to 35% for fine-grained sands.^19^

The study in^20^ points out that the areas where CO_2_ hydrate is stable are the polar regions and shallow water zones. As expected, salinity plays an important role in the stability of the CO_2_ hydrate and should not be neglected. The high geothermal gradient (more than 200 °C/km) inhibits the stability of CO_2_ hydrate and limits the areas of interest.^20^

New research has focused on counteracting the inhibiting effect of salinity, for instance, through the use of hydrophobic amino acids, which have been shown to significantly enhance hydrate formation rates. In particular, l-methionine has been reported to increase hydrate formation by up to 2.5 times by improving water structuring and reducing the energy barrier for CO_2_ diffusion by approximately 70%, thereby facilitating gas–liquid mass transfer.^21,22^

As already discussed for NGH and stated in^20^ the formation and persistence of CO_2_ hydrate in the marine environment, however, also depend on site-specific petrophysical properties of the porous medium, such as porosity, granulometry and mineralogy. Consequently, understanding how these local geological and thermodynamic conditions govern the CO_2_ hydrate behavior is key to evaluating the feasibility and optimization of this emerging storage pathway.

The present study is framed within this research context and aims to investigate the combined effects of salinity and natural sand on CO_2_ hydrate behavior. The combined influence of salinity and porous media on gas hydrate formation has been partially examined in the literature, mainly in the context of CH_4_–CO_2_ mixed hydrates in natural gas hydrate exploitation scenarios, indicating that porous media can significantly modify hydrate equilibrium by partially compensating for salinity inhibition.^23^ Nevertheless, while salinity is well-known to exert an inhibitory effect and porous media such as sand generally promote hydrate formation, pore-scale behavior differences during formation between CH_4_ and CO_2_ are reported^24^ and therefore it remains unclear how these two factors interact when both are present and affect pure CO_2_ hydrate formation in natural environments.

The future research directions outlined by Mehranjani et al.^25^ emphasize the need for integrated thermodynamic, kinetic and pore-scale approaches to understand CO_2_ hydrate behavior under realistic reservoir conditions.

In this framework, this study provides an experimental contribution by addressing some of the key physical factors identified in the literature (salinity and natural porous media) under controlled conditions representative of marine environments. An experimental framework was developed based on a direct comparison between binary (gas–water) and three-phase (gas–water–sand) systems, with and without the addition of salt. The analysis combines global performance indicators with kinetic and stability measurements, providing a comprehensive assessment of CO_2_ hydrate formation, dissociation and equilibrium behavior in salt- and sand-containing environments.

## 2 Materials and Methods

This section presents the equipment and materials employed, along with a detailed description of the experimental procedure. The setup is used to investigate CO_2_ hydrate formation and dissociation in a controlled laboratory environment, reproducing conditions found in marine sediments or waters. The experimental campaign investigates the formation, dissociation and stability of CO_2_ hydrates under various conditions (with and without sand, with and without salt).

### 2.1 Equipment

The experimental campaign is carried out using a 1 L reactor, equipped as shown in the schematic of the setup (Figure 1).

**Figure 1:**
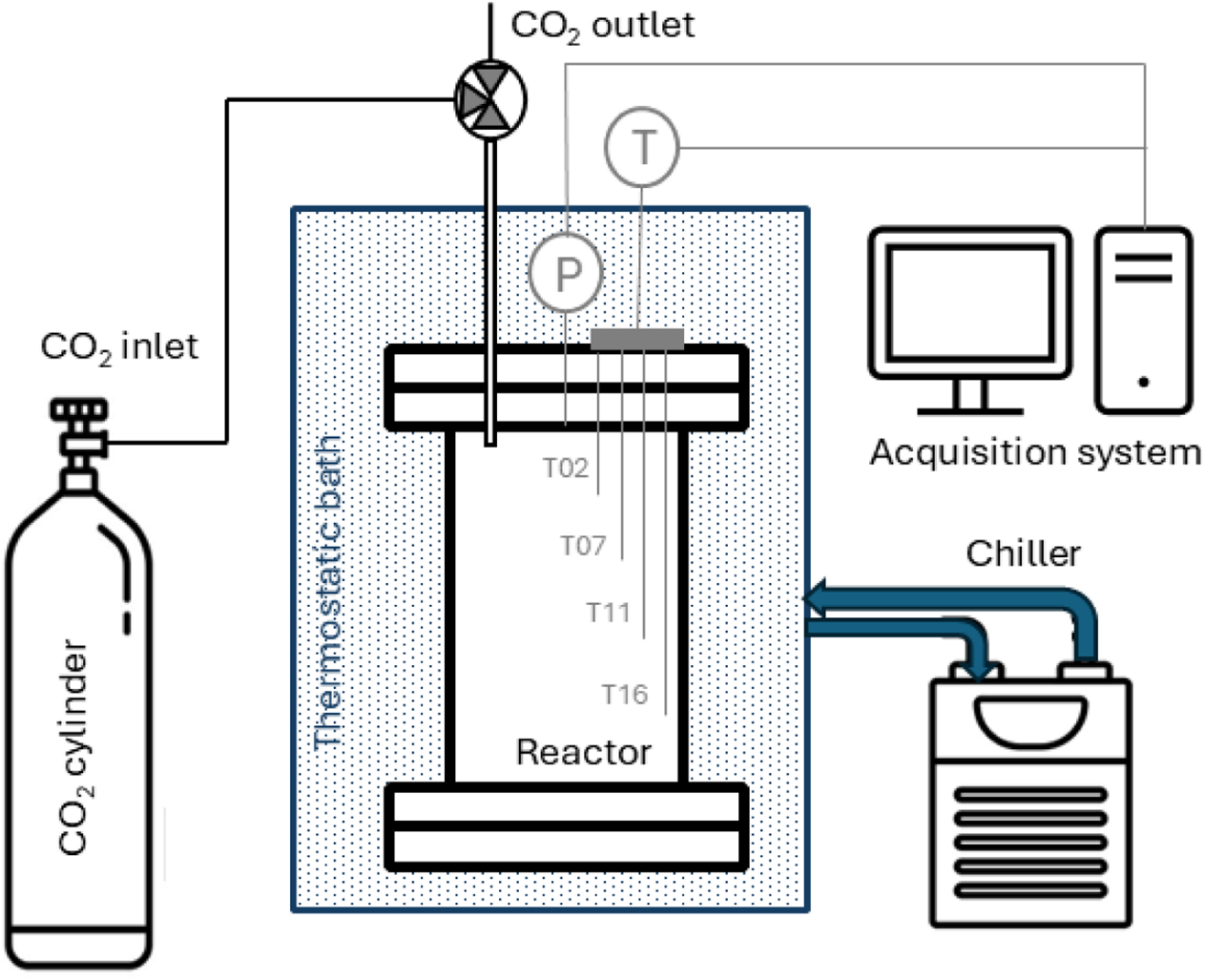
Schematic of the experimental setup.

CO_2_ is supplied from a gas cylinder and introduced into the reactor, which is previously filled with water and a porous medium to simulate natural environments. The reactor has a length of 21 cm and an internal diameter of 7.4 cm. It is equipped with a pressure sensor and four thermocouples positioned at different heights to continuously monitor the pressure and temperature during the experiment. As for the thermocouples, T02, T07, T11 and T16 are the numbers referring to depths in centimeters measured from the upper flange. To ensure stable and uniform thermal conditions, the reactor is placed inside a thermostatic bath cooled by a chiller (Model GCLT, Eurochiller) that circulates the coolant through an immersed serpentine coil. Throughout the experiments, the temperature and pressure data are continuously recorded by a data acquisition system connected to a computer.

### 2.2 Materials

In this experimental campaign, the reactor is filled either with a binary system (water and gas) or with a three-phase system (porous medium, water and gas).

In the binary system, the reactor is filled with water to 50% of its total volume. The water used is either pure distilled water or saline water (prepared by adding 3 wt % NaCl to distilled water). The NaCl concentration of 3 wt % was selected as a representative salinity value for marine environments. Although slightly lower than the average open-ocean salinity (3.5 wt %), it falls within the range commonly observed in coastal and semi-enclosed marine systems.^26^ In particular, in the Adriatic Sea, salinity can be reduced due to river discharge, especially in the northern basin.^27^ Lower salinity values (3.0–3.4 wt %) are also typical of polar regions, where freshwater contributions from ice melting further decrease seawater salinity.^28^

In the three-phase system, the reactor is filled with natural sand to 50% of its volume, then saturated with water (either pure or saline, containing 3 wt % NaCl) and CO_2_. CO_2_ bottles are supplied by Nippon Gases and have a purity of 99.95%.

The natural sand, sampled on the Adriatic Sea, has been morphologically characterized by Field Emission Scanning Electron Microscopy (FE-SEM, Sigma 300, Zeiss) operating at 20 kV and equipped with Energy Dispersive X-ray Spectroscopy (EDX, Quantax, EDS, Bruker). The porosity and the total volume of the pores of the natural sand were measured by BET measurements through volumetric N_2_ adsorption at −195.8 °C using an ASAP 2020 (Micrometrics) instrument.

The sand is formed mainly of quartz (SiO_2_) with traces of calcium carbonate and minor elements such as manganese, iron, and chromium. BET analysis indicates a surface area and total pore volume of 1.51 m^2^ /g and 0.0038 cm^3^ /g, respectively. The material is primarily mesoporous with limited microporosity: the micropore volume is 1.46 × 10^–4^ cm^3^/g and the micropore area is 0.34 m^2^/g. The average pore diameter is about 16 nm. SEM observations show some heterogeneity in particle size and surface morphology (Figure 2). From Figure 2, the particle size is mainly included in the range of 100–200 μm. This natural sand is representative of shallow marine sandy sediments, such as those typically found in continental shelf and upper slope environments. Also considering that the sand is saturated with water and the system is pressurized in the range of 25–35 bar, conditions corresponding to water depths of approximately 250–350 m are reproduced, which are characteristic of shallow offshore settings.

**Figure 2:**
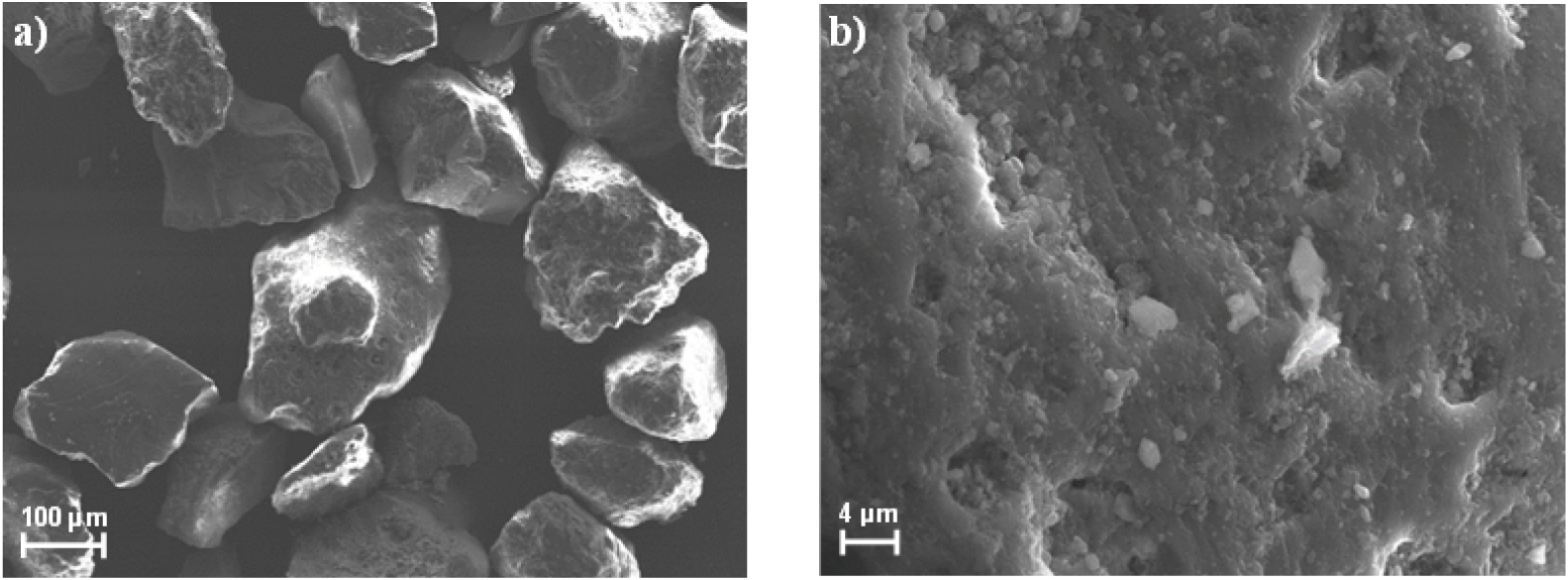
SEM images of the sand at different magnifications. (a) visible grains; b) surface of the grain).

### 2.3 Experimental Procedure for Hydrate Formation

For the formation tests, the reactor is first filled with water or with a mixture of sand and water, and then cooled to the initial temperature and pressurized with CO_2_ to reach the target initial pressure. The system evolves under isochoric conditions until hydrate formation is complete, while the evolution of pressure and temperature (p–T) values is continuously recorded. For every formation test, some numerical parameters are calculated to compare the effect of the different conditions; the definitions are given in Table 1. The first two calculated parameters are the uploaded CO_2_ moles and the CO_2_ moles entrapped in the formed hydrates. The gas uptake (G.U.) represents the efficiency of hydrate formation in terms of utilization of the available gas volume within the reactor. The formation density is the mass of CO_2_ stored per unit volume of water, providing an indication of the hydrate phase’s storage capacity. The water conversion rate (WCR) represents the percentage amount of water converted into hydrate and is the overall efficiency of the water-to-hydrate transformation process. These parameters provide a comprehensive assessment of the hydrate formation behavior and systemic performance under the tested conditions.

**Table 1:** Performance Parameters Evaluated during Hydrate Formation

Parameter	Equation	Calculation
Total number of CO_2_ moles injected into the reactor	$n_{CO_{2}}=\frac{P_{i}\cdot V_{\mathrm{free}\mathrm{gas}}}{Z_{i}\cdot R\cdot T_{i}}$	Determined from the free gas volume inside the reactor (*V*_freegas_), pressure and temperature of the free gas phase at the initial time of formation (*P_i_* and *T_i_*), applying the compressibility factor (*Z_i_*)
Number of CO_2_ moles absorbed in the formed hydrates	$n_{CO_{2}\mathrm{hyd}}=\frac{V_{\mathrm{free}\mathrm{gas}}\cdot \left(\frac{P_{i}}{Z_{i}\cdot R\cdot T_{i}}-\frac{P_{f}}{Z_{f}\cdot R\cdot T_{f}}\right)}{1-\frac{P_{f}}{Z_{f}\cdot R\cdot T_{f}\cdot \rho_{\mathrm{hyd}}}}$	Calculated from the change in gas pressure and temperature between the initial (*P*_i_, *T*_i_, *Z*_i_) and final states (*P*_f_, *T*_f_, *Z*_f_) of hydrate dissociation. This difference reflects the amount of CO_2_ consumed during hydrate formation.^a^
Gas uptake	$G.U.=\frac{n_{CO_{2}\mathrm{hyd}}}{n_{CO_{2}}}\times 100\left[\%\right]$	Calculated as the ratio between the moles of CO_2_ trapped in the hydrate phase and the total moles of gas injected, reported as a percentage.
Formation density	$$\rho_{\mathrm{form}}=\frac{n_{CO_{2}\mathrm{hyd}}\cdot M_{CO_{2}}}{V_{H_{2}O}}\left[g/m^{3}\right]$$	Obtained by multiplying the moles of CO_2_ in the hydrate by the molar mass of CO_2_ and dividing by the volume of water initially present in the system $\left(M_{H_{2}O}\right)$ .^b^
Water conversion rate	$WCR=\frac{n_{CO_{2}\mathrm{hyd}}\cdot n_{\mathrm{hydration}}}{\left(V_{H_{2}O}\cdot \rho_{H_{2}O}/M_{H_{2}O}\right)}\times 100\left[\%\right]$	Calculated as the amount of water involved in the formation of gas hydrates, considering the hydration number (5.75) and the amount of water available in the vessel, reported as percentage.^c^

a *ρ*_hyd_ is the CO_2_ hydrate density.

b $M_{CO_{2}}$ is the CO_2_ molar mass.

c $\rho_{H2O}$ is the water density, and $M_{H_{2}O}$ is the water molar mass.

To determine the kinetics of formation, the cumulative curves of the absorbed CO_2_ moles were built by applying the same formulation used for the calculation of the total number of CO_2_ moles in the formed hydrates (Table 1) to successive time intervals during the hydrate formation stage. At each time step, the moles of CO_2_ encapsulated in the hydrate phase were calculated by using the instantaneous pressure and temperature values, together with the corresponding compressibility factor. In this approach, the cumulative amount of CO_2_ absorbed at a certain time is obtained directly from the difference between the initial gaseous CO_2_ moles and those remaining in the gas phase at that specific time.

### 2.4 Experimental Procedure for Hydrate Dissociation

After formation, for the determination of the dissociated moles, the system was depressurized to atmospheric pressure, and cooling was stopped, allowing the hydrates to dissociate freely. During dissociation, the evolution of p–T values was monitored to evaluate the dissociation kinetics and to quantify the moles of CO_2_ released over time.

Similarly, for dissociation, the cumulative CO_2_ dissociation curves were constructed by accounting for the variation in both pressure and temperature, and for the progressive release of gas previously stored in the hydrate phase. The moles of CO_2_ dissociated between the beginning of dissociation and a generic time were calculated by using the analytical expression in Equation 1:

1 $$n_{\mathrm{diss}}=\frac{V_{g,0}\cdot \left(\frac{P_{1}}{Z_{1}\cdot R\cdot T_{1}}-\frac{P_{0}}{Z_{0}\cdot R\cdot T_{0}}\right)}{1-\frac{P_{1}}{Z_{1}\cdot R\cdot T_{1}\cdot \rho_{\mathrm{hyd}}}}$$

Where *P*_0_, *T*_0_, *Z*_0_ and *P*_1_, *T*_1_, *Z*_1_ are the pressure, temperature and compressibility factor at the beginning of dissociation and at a consequent time *t*_1_, respectively. The initial gas volume *V*_g,0_ corresponds to the free gas volume available at the beginning of dissociation (and thus at the end of formation) and is defined as in Equation 2:

2 $$V_{g,0}=V_{\mathrm{free}\mathrm{gas}}-\rho_{\mathrm{hyd}}\cdot n_{CO_{2}\mathrm{hyd}}$$

With *V*_freegas_ being the volume of free gas inside the reactor, ρ_hyd_ the hydrate molar density, and $n_{CO_{2}\mathrm{hyd}}$ the number of CO_2_ moles in the formed hydrates (Table 1).

This formulation was applied at each time step by substituting the instantaneous pressure and temperature values, thereby allowing the construction of cumulative dissociation curves that consistently track the progressive release of CO_2_ from the hydrates. Finally, time-averaged dissociation rates were calculated as discrete derivatives of the CO_2_ molecules released in each time step (Δ*n*/Δ*t*).

### 2.5 Experimental Procedure for Hydrate Equilibrium Tests

A second type of analysis was carried out to determine the equilibrium pressure–temperature (p–T) conditions of the hydrates under different environmental settings (salt and sediment) and to evaluate the combined effect of sediment and salinity on the equilibrium points. After the formation stage under isochoric conditions, the system was gradually heated while maintaining a constant volume, allowing the pressure to vary in response to temperature changes. Once hydrate formation was complete, the intersection of the dissociation and formation curves was identified as the phase equilibrium point of the gas hydrates under the given conditions. This procedure, known as the pressure search method, enables the identification of the equilibrium point.^29^

## 3 Results and Discussion

### 3.1 CO_2_ Hydrate Formation

The table summarizes the main experimental results from a series of CO_2_ hydrate formation tests conducted under varying conditions, comparing systems with and without natural sand and salt in the water. Eight tests are presented, related to four environmental conditions: pure water (PW), salt water with 3 wt % NaCl (3%SW), sand with pure water (PW+S), and sand with salt water (3%SW+S). For each condition, two initial pressures were defined (25 and 35 bar).

Table 2 reports, for each test, the initial and final pressures, the initial and final temperatures, the total moles of CO_2_ injected, the moles of CO_2_ entrapped in the hydrate phase and the key performance indicators (gas uptake, formation density, and water conversion rate).

**Table 2:** Results of the Experimental Tests

*N.*	*Name*	*Sand*	*NaCl*	*P*_*i*_ *(bar)*	*T*_*i*_ *(°C)*	*P*_*f*_ *(bar)*	*T*_*f*_ *(°C)*	$$n_{CO_{2}}$$	$$n_{CO_{2}\mathrm{hyd}}$$	*G.U. (%)*	*ρ*_form_ ( $g_{CO_{2}}$ /m^*3*^)	*WCR (%)*
1	PW (25 bar)	no	-	25.13	1.79	21.57	2.24	0.673	0.142	21.1	13.2	3.2
2	PW (35 bar)	no	-	34.56	3.16	18.00	2.19	1.635	1.377	82.6	125.1	30.8
3	3%SW (25 bar)	no	3%	25.64	2.3	24.24	2.66	0.688	0.059	8.6	5.5	1.3
4	3%SW (35 bar)	no	3%	34.56	2.88	30.03	2.37	1.683	1.088	64.6	100.7	24.7
5	PW+S (25 bar)	yes	-	25.00	1.84	16.69	0.09	0.217	0.094	43.2	36.5	9.0
6	PW+S (35 bar)	yes	-	34.77	2.21	28.8	–1.35	0.611	0.433	70.9	168.6	41.4
7	3%SW+S (25 bar)	yes	3%	24.69	2.52	19.6	1.54	0.212	0.060	28.5	23.5	5.8
8	3%SW+S (35 bar)	yes	3%	34.80	1.79	31.5	1.5	0.679	0.428	63.0	166.6	40.9

The values of the CO_2_ molecules captured in the hydrate phase clearly highlight the effect of salinity. The well-known inhibiting influence of salt (3 wt % NaCl) is confirmed, resulting in a lower amount of hydrate formed (tests 3–4 compared to tests 1–2), consistent with observations on saline systems reported in the literature. In fact, the electrostatic interactions between ions and water molecules, and the CO_2_ solubility reduction in salt water, reduce the formation of CO_2_ hydrates.^30,31^ One aspect that needs elucidation is the combined role of sand and salt and how the presence of sand affects salt inhibition. To this purpose, Figure 3 presents a comparative overview illustrating the values of gas uptake, formation density and water conversion rate across the four environmental conditions investigated, at the two pressure values of 25 and 35 bar. For all conditions, higher pressure enhances the hydrate formation performance. As an effect of the inhibition due to salt, the lowest value for all three parameters is observed for the binary system with salt water (3%SW).

**Figure 3:**
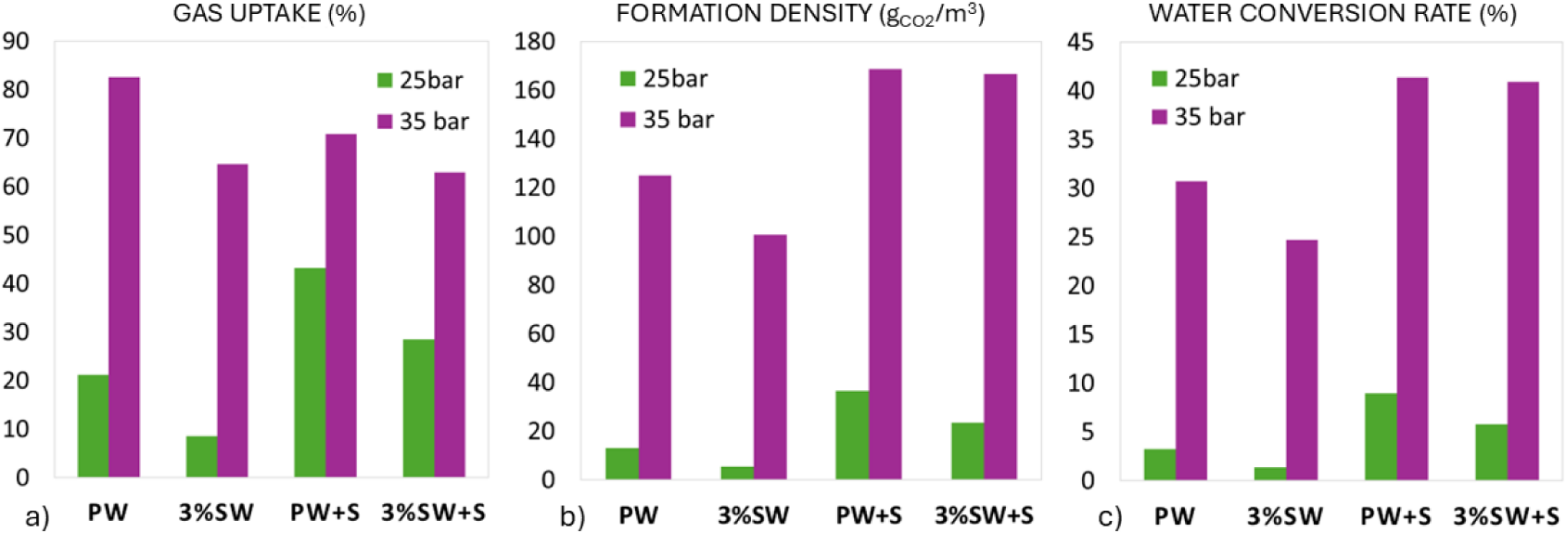
Performance parameter values in the four environmental conditions: gas uptake (a), formation density (b), and water conversion rate (c).

As far as gas uptake is concerned, it is higher in pure water systems (PW and PW+S) compared to salt systems (3%SW and 3%SW+S) at both pressures. Natural sand enhances gas uptake, both with and without salt, except for the PW test at 35 bar (Test 2 in Table 1) which exhibits the highest gas uptake value. This behavior can be attributed to the combined effect of high pressure and the absence of salinity. At 35 bar, the increased driving force enhances the dissolution of CO_2_ in the aqueous phase and promotes rapid mass transfer. In pure water, the activity of water is not reduced by dissolved ions, allowing a larger fraction of the injected CO_2_ to participate effectively in hydrate formation. As a result, the moles of CO_2_ trapped in the hydrate phase represent a higher fraction of the total CO_2_ injected, leading to the maximum gas uptake value, which, in fact, represents the efficiency of CO_2_ utilization relative to the amount of gas injected.^32^ The absence of salt-related penalties, therefore, enables optimal gas utilization efficiency at higher pressure.

Conversely, the formation density and water conversion rate quantify the efficiency of hydrate accumulation per unit volume of water and the extent of water-to-hydrate transformation, both of which are strongly governed by the presence of the porous medium. There is also a dependence on pressure.

For all systems, the formation density increases markedly at 35 bar, indicating a larger accumulation of CO_2_ hydrate within the water volume at higher pressure. The lowest formation densities are obtained in the saline binary system (3%SW), while the highest value is for pure water with sand (PW+S). The presence of sand leads to a substantial increase in formation density; in fact, the highest values of the formation density occur in PW+S and 3%SW+S at 35 bar (168.6 g_CO2_/m^3^ in Test 6 and 166.6 g_CO2_/m^3^ in Test 8).

Water conversion rate follows a trend similar to the formation density. At 25 bar, water conversion remains limited in all systems, particularly in binary systems (PW and 3%SW). Increasing the pressure to 35 bar significantly enhances water conversion under all conditions. Also, the introduction of sand substantially increases the parameter. The highest water conversion rate is achieved in a three-phase pure system (PW+S) at 35 bar.

So, the highest values of formation density and water conversion rate are observed for the PW+S system at 35 bar. The properties of the natural sand used in this study are consistent with the mechanisms described in the literature regarding the influence of porous medium characteristics on CO_2_ hydrate formation. The BET and SEM characterizations indicate that the Adriatic Sea sand has a predominant mesoporous structure, with grain sizes in the range of 100–200 μm. Literature shows that sands with similar grain sizes enhances gas storage capacity.^31^

The sediment, in fact, promotes spatially distributed hydrate growth and more effective water utilization, leading to high formation density and water conversion. Consistent with this explanation, the analysis in^33^ shows that, at 100% initial water saturation, the grain-size range (70–140 μm) exhibits the highest water conversion to hydrate compared to finer sands. In addition, surface irregularities shown in Figure 2 enlarge the effective interfacial area, enhancing heat transfer during hydrate formation and limiting local dissociation, while the voids due to the surface roughness promote capillary water uptake, increasing CO_2_water interaction,^25,34,35^

The combined presence of salt and sand is particularly noteworthy; to highlight this phenomenon, it is important to compare 3%SW systems with 3%SW+S systems. In saline systems without sand (3%SW), all parameters exhibit the lowest values, while with the introduction of natural sand (3%SW+S), the values significantly increase, indicating that the sediment counteracts the inhibitory effect of salinity.

Comparing 3%SW and 3%SW+S systems, gas uptake increases from 8.6% to 28.5% at 25 bar (Tests 3 and 7) and is similar at 35 bar (64.6% in Test 4 and 63.0% in Test 8); formation density passes from 5.5 to 23.5 g_CO2_/m^3^ at 25 bar (Tests 3 and 7) and from 100.7 to 166.6 g_CO2_/m^3^ at 35 bar (Tests 4 and 8); WCR passes from 1.3% to 5.8% at 25 bar (Tests 3 and 7) and from 24.7% to 40.9% at 35 bar (Tests 4 and 8). The enhancement is particularly significant at 35 bar, indicating that the compensating role of sand becomes more effective as pressure increases.

In saline binary systems, hydrate formation is strongly inhibited due to reduced water activity and CO_2_ solubility,^36^ but when natural sand is introduced, the system behavior changes due to the provision of solid–liquid interfaces that promote heterogeneous nucleation, reducing the energetic barrier for hydrate formation even in saline environments.^37^ Moreover, hydrate growth within the pore structure leads to a spatially distributed formation mechanism, preventing hydrate development from being confined to a limited number of interfaces. This effect is particularly relevant for formation density and water conversion rate, which depend on how effectively the available water is converted into hydrate rather than on the total amount of gas injected.

The incremental beneficial effect of sand differs depending on whether the aqueous phase is pure or saline. There is also a dependence on pressure. At 25 bar, the introduction of sand leads to a pronounced enhancement of both the formation density and water conversion rate. In pure water systems, formation density increases from 13.2 to 36.5 g_CO_2__/m^3^ and WCR from 3.2% to 9.0% (Tests 1 and 5), corresponding to nearly a 3-fold increase. An even stronger effect is observed under saline conditions, where formation density rises from 5.5 to 23.5 g_CO_2__/m^3^ and WCR from 1.3% to 5.8% (Tests 3 and 7), i.e., by more than a factor of 4. At this lower pressure, hydrate formation with salt and in the absence of sediment is strongly limited, and the porous medium plays a greatly enabling role.

In contrast, at 35 bar, the effect of sand becomes less important but remains clearly beneficial. In pure water, formation density increases from 125.1 to 168.6 g_CO_2__/m^3^ and WCR from 30.8% to 41.4% (Tests 2 and 6), corresponding to an increase of about one-third. Under saline conditions, the presence of sand enhances formation density from 100.7 to 166.6 g_CO_2__/m^3^ and WCR from 24.7% to 40.9% (Tests 4 and 8), i.e., by about two-thirds. This behavior indicates that, as pressure increases, the role of sand shifts from enabling to enhancing hydrate formation, with a more pronounced impact when salinity penalizes hydrate growth in bulk water.

### 3.2 Kinetics of Formation

Figure 4 reports the temporal evolution of cumulative CO_2_ absorbed during the first 240 min of hydrate formation experiments at 25 and 35 bar, and also includes a bar chart providing a time-integrated indicator of early-stage hydrate growth. The reported curves are limited to the first 240 min in order to highlight the initial and intermediate stages of hydrate formation.

**Figure 4:**
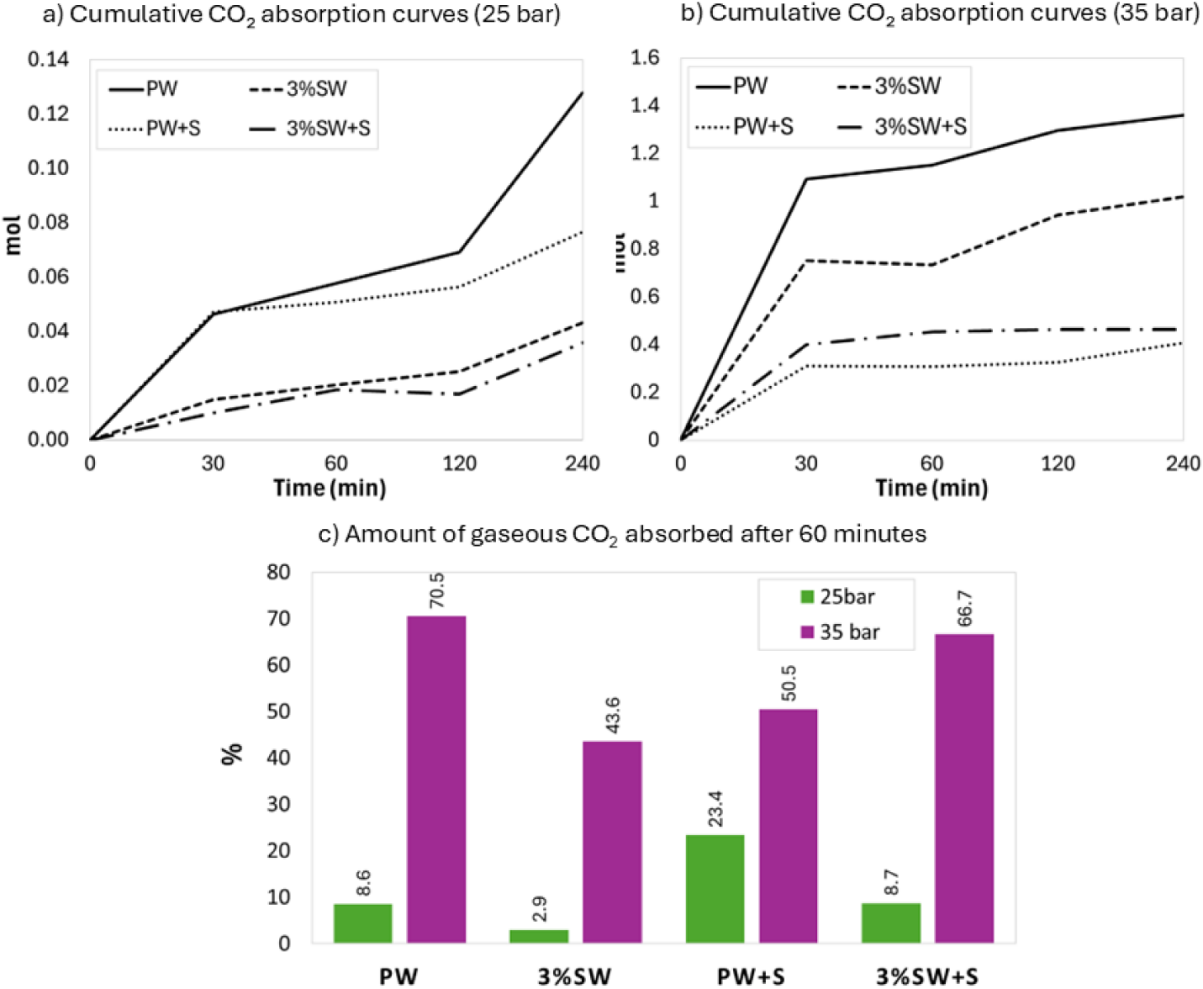
Cumulative CO_2_ absorption curves as a function of time at 25 and 35 bar for pure water (PW), saline water (3%SW) and the corresponding sand-containing systems (PW+S and 3%SW+S) (a,b), together with the percentage amount of gaseous CO_2_ absorbed into hydrates after 60 min (c).

At 25 bar (Figure 4a), the cumulative CO_2_ uptake increases gradually over time in all four systems, with only a marked difference for the PW system, which shows a rapid initial increase in absorbed CO_2_, followed by sustained growth over time. Salt systems (3%SW and 3%SW+S) exhibit a gradual and continuous increase in cumulative CO_2_ uptake over time but with lower values compared to the corresponding pure water systems (PW and PW+S), reflecting the inhibiting effect of salinity under low driving force conditions. The PW+S system exhibits an initial absorption trend close to that of pure water, followed by a slower increase. However, when considering the percentage amount of CO_2_ absorbed after 60 min as a time-integrated kinetic indicator (Figure 4c), the value for PW+S at 25 bar (23.4%) is higher than that of PW (8.6%). Similarly, the 3%SW+S system shows a cumulative behavior comparable to the 3%SW system over time, but the percentage of CO_2_ absorbed after 60 min is higher for the sand-containing system (8.7% versus 2.9%). This confirms that, under low-pressure conditions, the porous medium plays an enabling role in saline systems, also from a kinetic point of view, without introducing significant mass transfer limitations.

At 35 bar (Figure 4b), the pure water system (PW) exhibits a pronounced behavior, with a sharp increase within the first 30 min, indicating fast CO_2_ encapsulation and leading to the highest gas uptake value reported in Table 2 (Test 2). In addition, the differences between binary systems and three-phase systems become more evident. While in the PW and 3%SW systems, the cumulative CO_2_ uptake increases steadily over time, sand-containing systems (PW+S and 3%SW+S) exhibit two distinct kinetic regimes: an initial rapid increase up to approximately 30 min, followed by a plateau. In the presence of sand, initial hydrate growth and pore filling progressively introduce mass transfer limitations to gas diffusion within the porous structure. The PW system benefits from the high driving force, which sustains rapid hydrate growth, leading to the highest cumulative uptake, with 70.5% of the injected CO_2_ converted into hydrate after 60 min. When comparing PW and PW+S, the percentage of CO_2_ incorporated into hydrates after 60 min decreases from 70.5% to 50.5%. This behavior is consistent with literature findings, where hydrate growth within the pore network progressively reduces gas permeability and shifts the process from a gas-driven regime to a pore-controlled growth mechanism.^38^ At 35 bar, the amount of CO_2_ incorporated into hydrates after 60 min increases from 43.6% in the 3%SW system to 66.7% in the 3%SW+S system, highlighting the beneficial kinetic effect of sand in saline systems under high driving force conditions.

These experimental results can be further explained through insights from molecular dynamics simulations. In particular, hydrate growth has been shown to be controlled by CO_2_ availability and transport within the aqueous phase, with diffusion limitations leading to localized depletion and reduced growth rates.^22^ In addition, hydrate formation preferentially occurs at the hydrate-water interface, where the progressive consumption of nearby water molecules can further limit growth as the reaction proceeds.^39^

In saline systems, the presence of ions interferes with hydrate cages during growth, contributing to fluctuations in cage formation and decomposition.^39^ This is consistent with spectroscopic evidence showing that hydrate nucleation is associated with an increase in hydrogen-bond ordering in water, while dissolved salts tend to inhibit this process by disturbing the hydrogen-bond network.^21^

Within this framework, the results of this work suggest that the presence of sand mitigates the limitations brought by salinity. The sand provides additional nucleation sites and enhances CO_2_ redistribution within the pore space during the early stage of formation when the gas–liquid contact is dominant. Moreover, surface silanol groups (Si–OH) on quartz can interact with water molecules through hydrogen bonding, further facilitating heterogeneous nucleation and interfacial organization.^40^

These interpretations support the existence of an interplay between salinity and sand, where molecular-scale inhibition is compensated for by pore-scale interfacial processes, controlling hydrate formation kinetics and overall conversion.

### 3.3 Dissociation Kinetics

Figure 5 shows the cumulative amount of CO_2_ released during hydrate dissociation as a function of time for the four investigated systems (PW, 3%SW, PW+S, and 3%SW+S) at formation pressures of 25 and 35 bar, and the dissociation-rate analysis expressed in terms of average Δ*n*/Δ*t* over successive time intervals.

**Figure 5:**
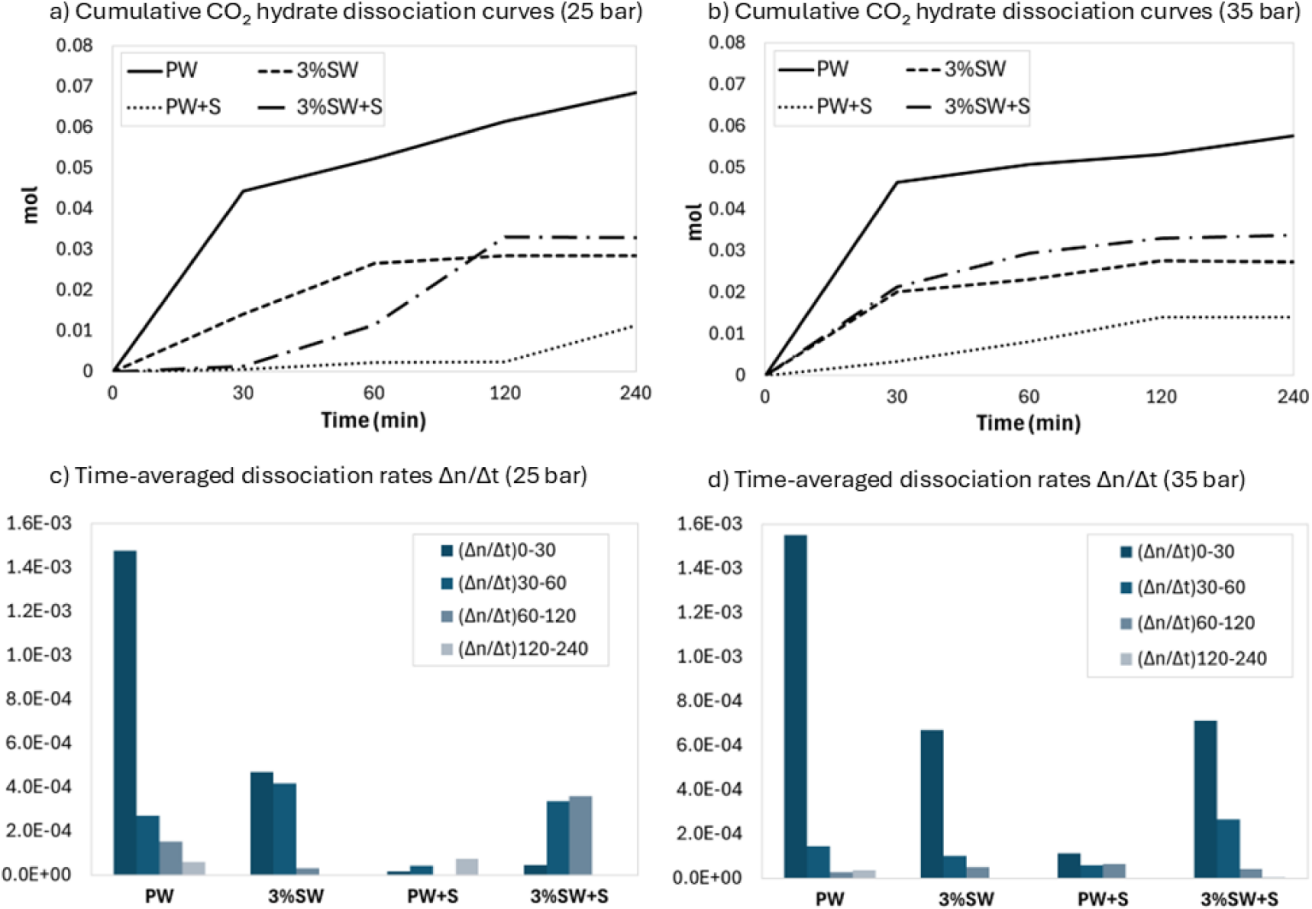
Cumulative dissociated CO_2_ moles as a function of time at 25 and 35 bar for pure water (PW), saline water (3%SW) and the corresponding sand-containing systems (PW+S and 3%SW+S) (a,b), together with time-averaged dissociation rates (Δ*n*/Δ*t*) of CO_2_ hydrates at 25 and 35 bar (c,d), calculated over successive time intervals (0–30, 30–60, 60–120, and 120–240 min).

At 25 bar (Figure 5a), in the PW system, dissociation is characterized by a rapid initial release in the first 30 min, followed by a slower but continuous increase up to 240 min. In the 3%SW system, the initial dissociation rate is lower than in pure water, but dissociation proceeds steadily until a plateau is reached after approximately 120 min. The early stabilization of the cumulative curve suggests that the limited formed hydrates dissociate relatively quickly, completing the process at early times. A different behavior is observed when natural sand is present. In the PW+S system, dissociation is delayed: the cumulative curve remains very low over the entire time interval, with only a slight increase after 120 min. The 3%SW+S system exhibits an intermediate but distinctive behavior. Dissociation is initially very limited, followed by a delayed but more pronounced increase between 60 and 120 min, before approaching a plateau. Compared with the binary saline system, the presence of sand leads to a slightly higher cumulative amount of CO_2_ released at long times, reflecting the larger amount of hydrate previously formed. However, the delayed onset and smoother slope indicate a more controlled dissociation process.

At 35 bar (Figure 5b), the PW system exhibits the highest cumulative amount of CO_2_ released over the entire observation period. The curve shows a steep initial increase, followed by sustained and linear growth. In the PW+S system, the cumulative dissociation curve is again the lowest one. Despite the higher formation density measured during hydrate formation, the dissociation proceeds slowly and remains limited throughout the experiment. The 3%SW system displays a lower curve compared to PW. Both 3%SW and 3%SW+S curves show an initial increase, followed by a progressive slowdown, approaching a plateau at longer times. Nevertheless, the 3%SW+S curve lies slightly above that of 3%SW, reflecting the larger amount of hydrate previously formed in the presence of sediment rather than a change in intrinsic dissociation kinetics.

Figure 5c, d shows dissociation rates in different stages of the dissociation process. Bulk systems (PW and 3%SW) exhibit the highest dissociation rates in the early stage, while sand-containing systems in pure water have the lowest dissociation rates. The only notable difference between the two pressures concerns the saline sand system (3%SW+S), which shows two opposite trends. At 25 bar, the dissociation rate increases in the last stages, while at 35 bar, the dissociation rate is higher at the beginning and then decreases. At lower pressure, limited hydrate formation leads to delayed but increasing dissociation rates at longer times, whereas at higher pressure, the larger amount of formed hydrates results in higher initial dissociation rates followed by transport-controlled decay. Therefore, the observed behavior reflects more on formation history and hydrate morphology rather than changes in intrinsic stability.

The kinetic analysis of dissociation is the basis for interpreting the effect of pressure, salt and sand on it. Formation pressure indirectly controls dissociation kinetics through the amount of hydrate formed during the previous formation stage.

Salinity leads to a fluctuating hydrate formation process, with competing formation and dissociation processes;^39^ consequently, saline bulk systems (3%SW) exhibit initial significant dissociation rates followed by a plateau compared to PW systems (Δ*n*/Δ*t* = 0 for 120–240 min, Figure 5c,d).

Sand in pure water brings the lowest dissociation rates, even with high hydrate occurrence. This confirms what is found in the literature: dissociation behavior is strongly controlled by the amount of hydrate formed within the sediment, so that higher hydrate occurrence results in slower dissociation.^41^ In addition, the absence of salt results in a less fluctuating formation regime and spatially distributed hydrates that dissociate in a controlled manner. When sand and salinity are present together, their effects on dissociation kinetics are nonadditive and pressure-dependent, as explained above.

### 3.4 Stability Measurements

Figure 6 illustrates the CO_2_ hydrate equilibrium conditions obtained for the different experimental systems, highlighting the influence of salinity and natural sand on hydrate stability. The pressure–temperature (p–T) equilibrium curves for pure water (PW) and saline water containing 3 wt % NaCl (3%SW) were calculated using the HydOff software, whereas the equilibrium points for PW+S and 3%SW+S were experimentally determined using the pressure search method.

**Figure 6:**
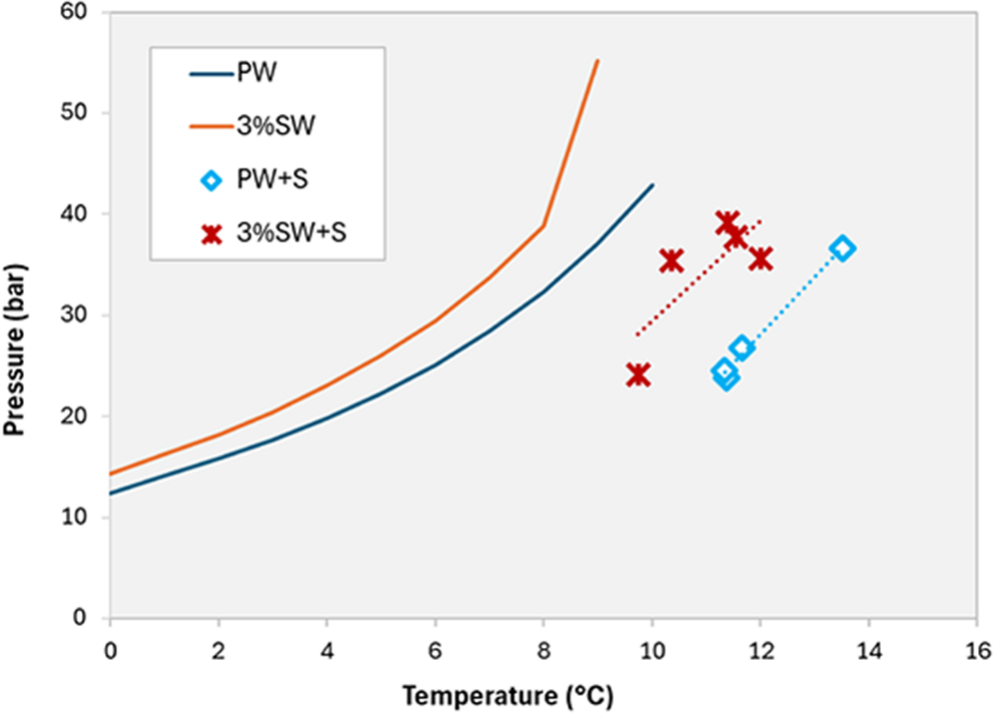
Pressure–temperature (P–T) equilibrium conditions for CO_2_ hydrates in pure water (PW), saline water (3%SW), and the presence of natural sand (PW+S and 3%SW+S).

Table 3 summarizes the initial experimental conditions used to build the equilibrium curves and the obtained points shown in Figure 6.

**Table 3:** Experimental Conditions and Results of the Equilibrium Tests

System	Initial pressure of formation P_iform_ (bar)	Initial temperature of formation T_iform_ (°C)	Equilibrium Pressure (bar)	Equilibrium temperature (°C)
PW+S	32.77	2.21	36.64	13.51
PW+S	34.30	6.90	36.64	13.51
PW+S	25.95	1.57	23.79	11.37
PW+S	23.90	0.73	24.56	11.34
PW+S	24.44	1.00	26.82	11.66
3%SW+S	24.69	2.52	24.15	9.74
3%SW+S	34.99	1.50	35.60	12.00
3%SW+S	35.00	1.79	39.13	11.39
3%SW+S	33.10	2.21	35.44	10.36
3%SW+S	34.63	3.78	37.79	11.54

The binary system curves show the well-known inhibitory effect of salinity on hydrate formation, as the equilibrium curve of the saline system (3%SW) is shifted toward higher pressures and lower temperatures relative to pure water.

In the presence of natural sand, the experimentally determined equilibrium points for both PW+S and 3%SW+S systems are shifted toward slightly higher temperatures at a given pressure with respect to the corresponding bulk systems. In such conditions, hydrate stability reflects the global response of spatially distributed pore-scale domains rather than a single homogeneous interface and is governed by gas–liquid–solid interfacial interactions, heterogeneous nucleation and local confinement effects.

The system with salt and sand (3%SW+S) exhibits intermediate behavior. When the 3%SW+S curve is compared with the PW+S curve, salinity continues to exert an inhibiting effect, as the 3%SW+S curve remains shifted to lower temperatures compared to PW+S. Nevertheless, the presence of sand partially compensates for this inhibition: the 3%SW+S curve is positioned to the right of the theoretical PW equilibrium curve, whereas the bulk saline system (3%SW) lies to the left. This indicates that porous-medium effects can mitigate the adverse impact of salinity, resulting in equilibrium conditions that are less restrictive than those predicted for bulk saline water.

Other results in the literature show condition-dependent effects of silica porous media on hydrate equilibrium. Giovannetti et al.^40^ showed that, for coarse quartz sands with particle sizes exceeding 200 μm, the results are not univocal, depending on salinity. In pure water systems, the presence of sand promotes hydrate stability compared to bulk water. Also, Uchida et al.^42^ observed that, for intermediate grain sizes of 100 μm and pure water, the hydrate equilibrium curve remains slightly shifted toward more favorable formation conditions. The present study, employing sand with particle sizes between 100 and 200 μm, is consistent with this behavior. For finer silica sands (particle sizes below 90 μm), Ndlovu et al.^29^ reported that the equilibrium curve intersects the corresponding bulk curve, exhibiting an inhibiting effect at lower temperatures and a promoting effect at higher temperatures. A similar intersection was also reported by Giovannetti et al.,^40^ but in the case of a sand-containing system with salt.

The comparison with the literature data outlines a framework linking sediment grain size to the equilibrium curves of CO_2_ hydrates. Intermediate grain sizes represent a transitional stable regime in which, probably, the confinement effects are limited, and the gas–liquid–solid interfacial area remains sufficiently large to promote hydrate formation and compensate for salinity-induced inhibition, without intersections.

Overall, salinity remains the primary parameter controlling the thermodynamic equilibrium of CO_2_ hydrates, while sediment grain size governs the porous medium effects that modulate the experimental equilibrium, determining whether the salinity inhibition is enhanced or attenuated.

## 4 Conclusion

This work investigated the individual and combined effects of salinity and natural sand on the formation, kinetics and stability of CO_2_ hydrates under conditions representative of marine environments. A systematic comparison between binary (gas–water) and three-phase (gas–water–sand) systems, with and without salinity, at two different levels of initial pressure (25 and 35 bar), was carried out using global performance indicators, kinetic analysis (in formation and dissociation), and equilibrium measurements.

Results demonstrate that the combined presence of salinity and natural sand leads to a nonadditive and pressure-dependent response in CO_2_ hydrate systems:

1. When natural sand is considered in saline systems, the inhibition caused by salt is significantly attenuated: in saline sand-containing systems, all performance indicators increase markedly compared to the saline binary system, with the enhancement becoming more pronounced at higher pressure. This confirms that sediment effectively counteracts the negative impact of salinity by promoting hydrate formation and more efficient water utilization.
2. Hydrate formation kinetics are primarily controlled by pressure, with salinity and sediment exerting secondary but condition-dependent effects. At low pressure, the cumulative absorption curves show similar overall trends, while salt and natural sand act in opposite directions: salinity suppresses hydrate growth, whereas the presence of sand facilitates early-stage formation, particularly in saline systems, acting as a kinetic enabler without introducing significant mass transfer limitations. At higher pressure, the higher driving force amplifies kinetic differences between bulk and sand-containing systems. Bulk systems sustain continuous CO_2_ uptake over time, while sand-containing systems exhibit a rapid initial uptake followed by a plateau in the cumulative curves, indicating a transition to pore-controlled growth. At higher pressures, sand increases the fraction of CO_2_ incorporated during the early stage in saline environments.
3. During dissociation, salinity primarily controls the dissociation rates, while sand redistributes hydrate within the pore space, modifying the temporal pattern of CO_2_ release. Bulk systems show higher initial dissociation rates at both pressures, while sand-containing systems without salt dissociate very slowly. In the specific case of the saline three-phase system, the behavior is pressure-dependent as a consequence of the extent of the previous hydrate formation and, at higher pressures, similar to the saline bulk system.
4. Equilibrium analysis shows that the presence of natural sand modifies the equilibrium conditions, shifting hydrate stability toward more favorable conditions and partially compensating for salinity inhibition. For the investigated grain-size range (100–200 μm), natural sand attenuates salinity penalties without inducing equilibrium curve intersections, identifying a stable regime that is favorable for hydrate-based CO_2_ storage.

The performed combined analysis demonstrates that salinity and sediment do not act independently but interact in a pressure-dependent manner. These results provide experimental evidence that natural sediments with intermediate grain size can mitigate salinity penalties and enhance hydrate-based CO_2_ storage efficiency and stability, supporting the feasibility of this approach in sediment-rich marine environments.
